# Root canal morphology of anterior permanent teeth in Jordanian population using two classification systems: a cone-beam computed tomography study

**DOI:** 10.1186/s12903-024-03934-2

**Published:** 2024-02-02

**Authors:** Nessrin A Taha, Nisrein Makahleh, Fatma Pertek Hatipoglu

**Affiliations:** 1 Department of Conservative Dentistry, Faculty of Dentistry, Jordan University of Science and Technology, Box 3030, Irbid, Jordan; 2 Dental Teaching Clinics, Jordan University of Science and Technology, Irbid, Jordan; 3 Department of Endodontics, Nigde Omer Halisdemir University, Nigde, Turkey

**Keywords:** Mandibular anterior teeth, CBCT, Root canal morphology, Vertucci classification, Ahmed classification

## Abstract

**Background:**

Adequate knowledge of root canal morphology and its variation is essential for success of root canal treatment and to overcome treatemnt failure. The aim of this study was to investigate the root and canal morphology of mandibular anterior teeth using 2 classification systems.

**Methods:**

3342 lower anteriors were evaluated from 557 CBCT scans. The images were examined in sagittal, axial and coronal views using a CS 3D imaging software (V3.10.4, Carestream Dental). Demographic data recorded, the number of roots and canal’s morphology were described according to Vertucci and Ahmed classifications.

**Results:**

Frequency of Type I configuration was significantly the highest in incisors and canines (76%, *N* = 2539), followed by Type III (20.6%, *N* = 687). Type II (1.1%, *N* = 37), IV (1.1%, *N* = 37), and V (0.3%, *N* = 11) were rarely encountered. 0.9% (*N* = 31) of the teeth could not be classified with the Vertucci System. The frequency of 2 roots (^2^MA in Ahmed classification) which has no correspondence in the Vertucci classification, was 1.1% (*N* = 38), it was significantly higher in canines and in females (35 canines and 3 laterals). A moderate correlation in root canal morpology was found between the left and right sides (V > 0.30). 80% (*N* = 2538) of the teeth did not exhibit any divergence/merging. The bifurcation level occurred mostly in the middle third of the root.

**Conclusions:**

One fourth of anterior teeth had variation from the simple type I canal configuration and therefore requires attention during treatment. The new classification system offers a more accurate and simplified presentation of canal morphology.

**Clinical relevance:**

The prevalence and mid root bifurcation of second canal in lower anteriors requires attention to ensure adequate quality root canal treatment without compromising the integrity of teeth.

## Background

Failure of endodontic treatment is often a concern for both clinicians and patients. The key to success lies in proper instrumentation, disinfection, and filling of the root canals. Procedural errors can arise from the lack of knowledge about anatomical variations in the root canal system, ultimately leading to treatment failure [1]. Therefore, adequate knowledge of the root and canal morphology is a prerequisite for successful endodontic treatment.

Studies have found that the number and structure of root canals differ among teeth [2, 3]. It has been reported that one or two canals are often present in mandibular anterior teeth (MDA) [4]. Wide variation in root canal morphology has been reported, particularly in the mandibular incisors (MDI) [3, 5]. A recent study among the Malaysian population reported that the MDA exhibit a wide range of canal variation, and canal complexity is significantly influenced by gender, ethnicity, and age [6]. Several studies among South African, Turkish, Malaysian, and American populations have revealed differences in root canal morphology of permanent anterior teeth [2, 5–7].

The Vertucci classification has been widely used since 1984 to describe root canal morphology [7]. However, some deficiencies in this system have been reported over time, such as the inability to identify two/three rooted teeth and the inability to classify various root canal systems [8, 9]. Due to these limitations in the original Vertucci system, additions were proposed by Sert, et al. in 2004 to describe more complex canal configurations [3], and in 2017 Ahmed, et al. developed a simple and useful alternative classification system in which a single descriptive code represents both canal morphology and root number [8].

Various techniques such as decalcification [10], dye injection [3], ex vivo radiography [11], in vitro macroscopy [12], scanning electron microscopy [11], cone-beam computed tomography (CBCT) [2], and micro-CT [13], have been used to study tooth anatomy. CBCT has become the imaging tool of choice in endodontic practice and in determining root canal morphology [14]. CBCT is non-invasive and helps to detect the complex external and internal anatomical structures of the tooth and nearby structures in details. It has a relatively low radiation dose and it is more economical than a CT imaging system. It has been shown that CBCT is a reliable tool for imaging root canal morphology and can be used for cross-sectional studies with a large sample size [15].

In the literature, there are a limited number of studies in which the morphology of MDA was determined by by Ahmed classification system [15, 16]. No data is available on root canal morphology of MDA among Jordanians using CBCT or Ahmed classification system. The aim of this study was to evaluate the root and canal morphology of permanent MDA in a Jordanian population using CBCT and to compare the findings based on 2 root canal classification systems (Vertucci and Ahmed classifications). The variation in root canal morphology in relation on gender and age was also analyzed.

## Methods

Ethics approval was obtained from the institutional review board. The sample size for this cross-sectional study was calculated with a 95% confidence level, 5% precision, and 50% expected prevalence (Maximized based on its unpredictability), at least 384 teeth (192 CBCT images) were planned to be included.

A retrospective analysis of all available CBCTs at a private CBCT imaging centre taken fin the period from January 2021 to January 2023 was done. The CBCT was not performed for any patient for the purpose of the study, images were taken by referrals for diagnosis, treatment or follow-up. Patients routinely signed a consent form at the centre for using threir images for research purposes whenever needed.

### Origin of scans

A single CBCT unit (Myray Hyperion X5 3D/2D version) acquired all included scans. The parameters of the CBCT machine were variable and adjusted as necessary at the time of acquisition as clinically indicated. The CBCT machine parameter ranges were as follows: resolution 80 μm, number of basic frames—300 to 750, tube current—4 to 14 mA, tube voltage—60 to 85 kV, focal spot diameter—0.6 mm, FOV 7 cm x 10 cm and 150 μm Voxil size.

### Inclusion and exclusion criteria

Lower jaw scans of mature anterior teeth including canines, lateral incisors and central incisors present bilaterally were included. Patient’s age above 17 years. Only scans of acceptable quality CBCT images covering the MDA, to visualise individual roots and canals as well as the entire pulp chamber and apex were included. Only scans from individuals classified as Jordanians, as determined by the centre data base, were included.

The exclusion criteria were: immature teeth with open apices, previous endodontic treatment, the presence of posts (metal or fiber-posts), large metal restorations, crowns, bridges obscuring the anatomy, evidence of previous or apical/periodontal surgery, extensive resorption, calcification, and scatter impeding proper visualisation.

### Analysis of scans

A total of 759 scans were initially assessed, and 557 scans were included according to the above inclusion and exclusion criteria. All teeth were analyzed in 3 planes (coronal, sagittal, and axial) to determine the root number and internal configurations of each tooth. The software used was CS 3D imaging V3.10.4. international version (Carestream Dental LLC, Atlanta, USA). Two experienced endodontists were involved in the evaluation of the included teeth. The examiners were calibrated prior to data collection by assessing 50 individual teeth, one examiner evaluated all the scans and the second examiner evaluated a 10% subset. Brightness, contrast and sharpness filters were adjusted as necessary to allow improved visualisation.

**Fig. 1:**
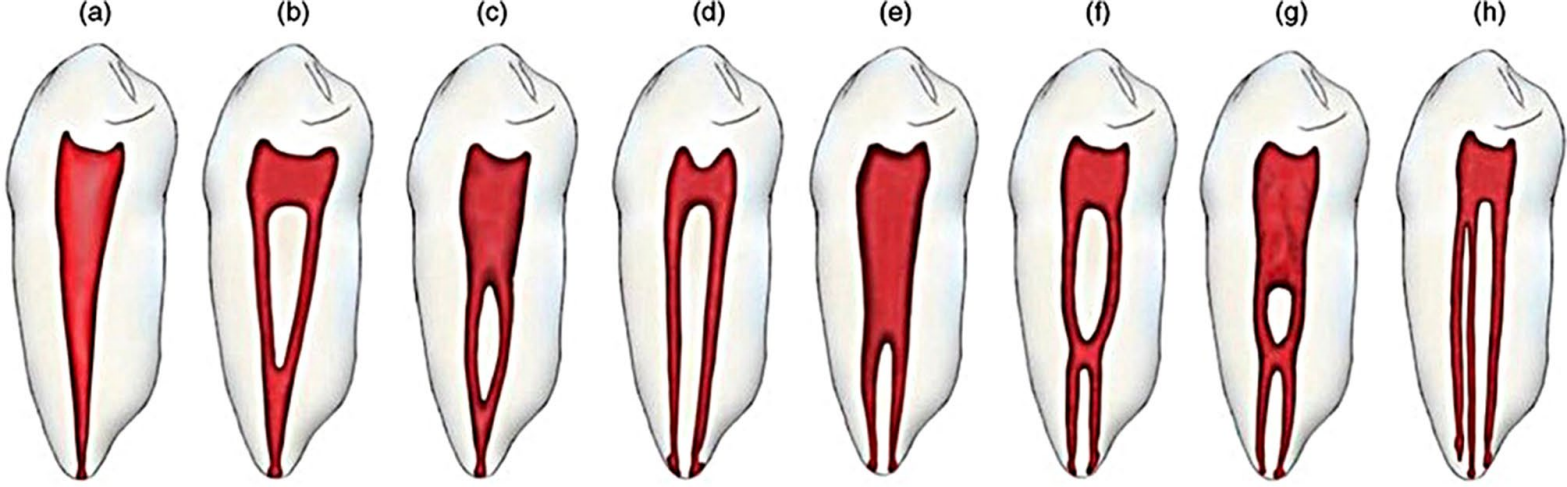
Vertucci’s classification of root canal morphology from type I to type VIII. (i) (**a**) Type I (1–1), (**b**) type II (2 − 1), (**c**) type III (1-2-1), (**d**) type IV (2–2), e) type V (1–2), (**f**) type VI (2-1-2), (**g**) type VII (1-2-1-2), (**h**) type VIII (3–3) (from Saber, et al. [18] with permission from Wiley)

**Fig. 2:**
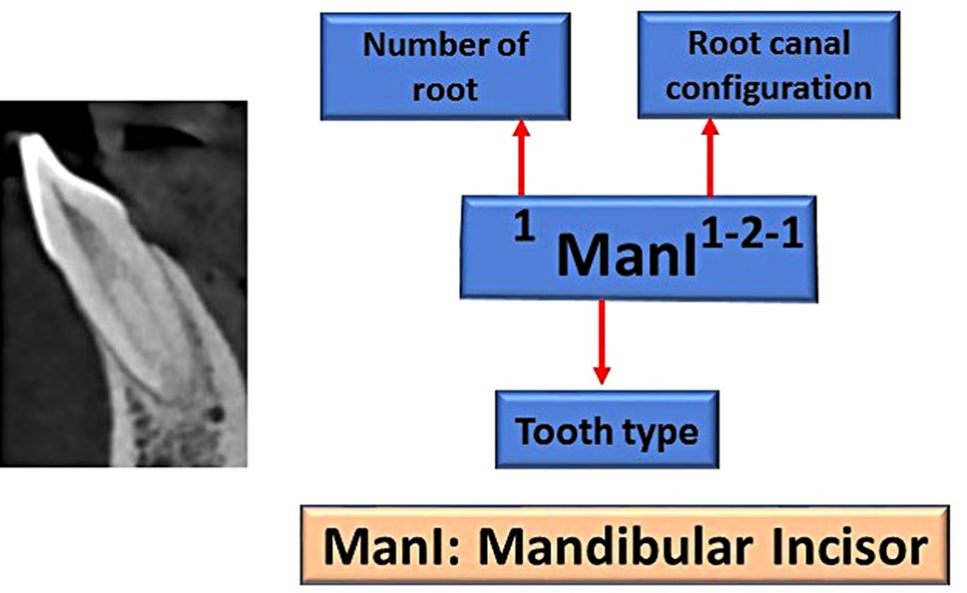
Ahmed classification system for root canal morphology of a mandibular incisor described with a code ^1^ManI^1–2−1^. The code consists of three components, the tooth code, number of roots added as a superscript before the tooth number, and the root canal configuration written as superscript after the tooth number

The obtained images were divided based on the patient’s age into 2 groups according to the mean age (36 years old and under, above 36 years). The root canal morphology was classified using Vertucci classification system (Fig. 1) and the new classification system introduced by Ahmed et al. [8] (Fig. 2), and the differences concerning age and gender were recorded.

### Number of roots

The number of roots and level of divergence and convergence were evaluated according to the following:

A single-rooted tooth: a tooth that had a clear single root. A double-rooted tooth: a tooth that had bifurcated roots (regardless of partial or complete root separation).

Diverging and merging levels of the roots and root canals: using the software ruler, each root was divided into three-thirds: a coronal section (from the cementoenamel junction to 1/3 of the root length), a middle section (from 1/3 to 2/3 of the root length) and an apical section (from 2/3 of the root length to the radiographic apex) [17].

### Statistical analysis

The Jamovi software (version: 2.3.21) was used for the statistical analysis. A descriptive analysis was conducted, and the differences in frequencies of the classifications according to tooth type were evaluated using a sample proportion test. The chi-square test was performed to examine the relationship between demographic characteristics and frequencies in the classifications. The differences between the tooth types with regard to the level of bifurcation and divergence/merging were examined using the chi-square test. Cramer’s V was used to examine the correlation between left and right sides. Significance was set at *p* < 0.05.

## Results

A total of 557 CBCT images were evaluated, 54% of the participants were female (*N* = 298) and the median age was 36 years (SD 10.84, IQR 17–67).

**Fig. 3:**
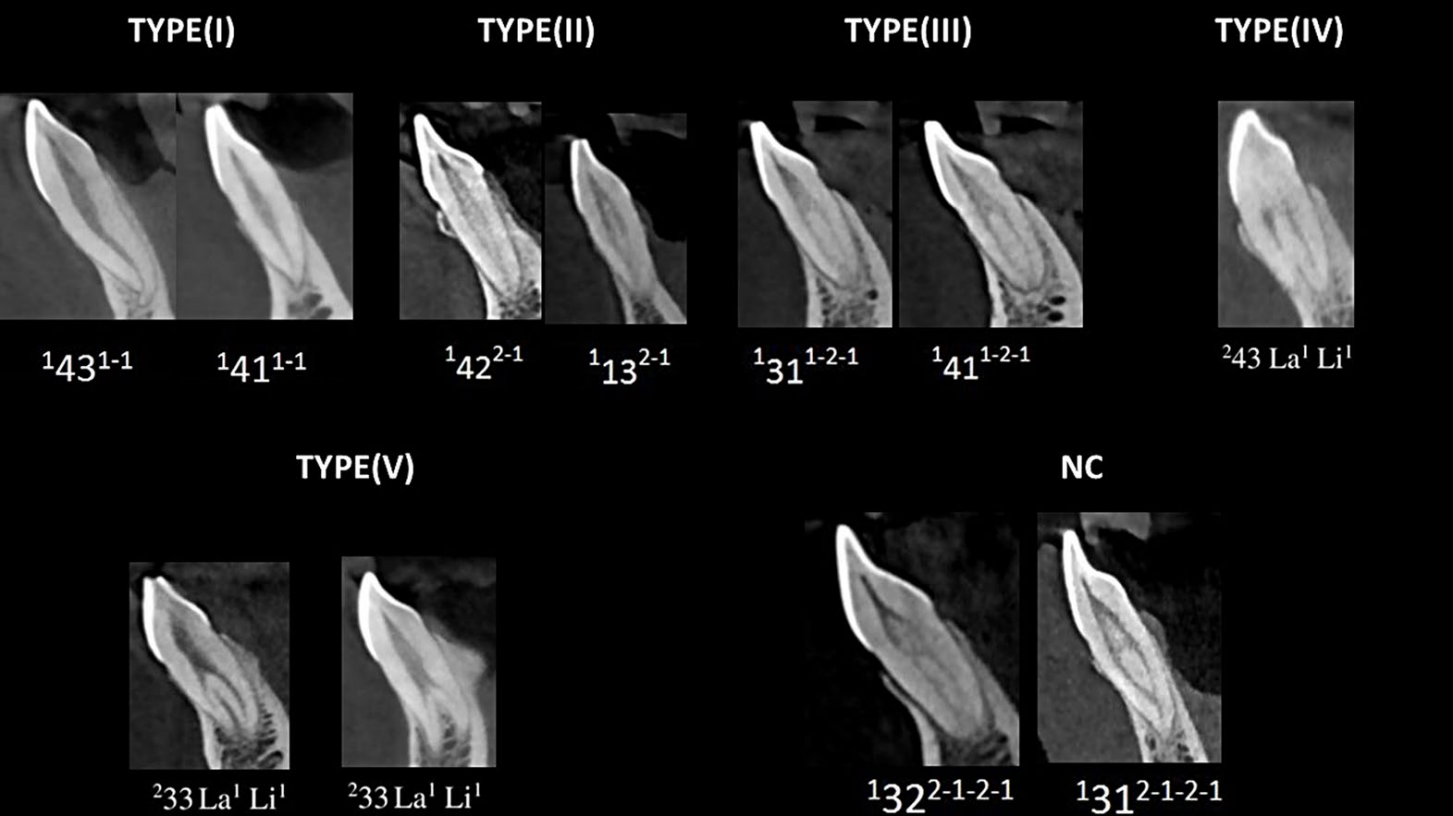
CBCT images demonstrating root canal morphological variations of mandibular anterior teeth using Vertucci and Ahmed classification

The frequency of Type I Vertucci was significantly the highest in all tooth types (76%, *N* = 2539), followed by Type III (20.6%, *N* = 687). Whereas type II (1.1%, *N* = 37), IV (1.1%, *N* = 37), and V (0.3%, *N* = 11) were rarely encountered (Fig. 3). There was a significant difference between tooth types regarding Type I, II, III, and IV of Vertucci classification (*p* < 0.05), but no siginificant difference was found for Type V (*p* > 0.05). While type I and IV were higher in canines (*p* < 0.05), other types were lower in canines (*p* < 0.05). 31 teeth (0.9%) could not be classified according to Vertucci classifiucation (Table 1; Fig. 3).

**Table 1:** Frequency of root canal configuration according to tooth type and 2 classification systems

	Central (*N* = 1114)	Lateral (*N* = 1114)	Canine (*N* = 1114)	Total (*N* = 3342)	p-value	
Vertucci classification						
I	787 (70.6%)	780 (70%)	972 (87.3%)	2539 (76%)	< 0.001^1^	
II	8 (0.7%)	7 (0.6%)	22 (2%)	37 (1.1%)	0.003^1^	
III	300 (26.9%)	310 (27.8%)	77 (6.9%)	687 (20.6%)	< 0.001^1^	
IV	0 (0%)	3 (0.3%)	34 (3.1%)	37 (1.1%)	< 0.001^1^	
V	3 (0.3%)	3 (0.3%)	5 (0.4%)	11 (0.3%)	0.695^1^	
NC	16 (1.4%)	11 (1%)	4 (0.4%)	31 (0.9%)	0.030^1^	
p-value	< 0.001^1^	< 0.001^1^	< 0.001^1^			
Ahmed classification						
^1^MI^1−1^	787 (70.6%)	779 (69.9%)	972 (87.3%)	2538 (75.9%)	< 0.001^1^	
^1^MI^1–2^	3 (0.3%)	3 (0.3%)	4 (0.4%)	10 (0.3%)	0.905^1^	
^1^MI^1–2−1^	300 (26.9%)	311 (27.9%)	77 (6.9%)	688 (20.6%)	< 0.001^1^	
^1^MI^2−1^	8 (0.7%)	7 (0.6%)	22 (2%)	37 (1.1%)	0.003^1^	
^1^MI^2−1−2−1^	16 (1.4%)	11 (1%)	4 (0.4%)	31 (0.9%)	0.030^1^	
^2^MI	0 (0%)	3 (0.3%)	35 (3.1%)	38 (1.1%)	< 0.001^1^	
p-value	< 0.001^1^	< 0.001^1^	< 0.001^1^			

^1^ One sample proportion test, NC: not classified

**Fig. 4:**
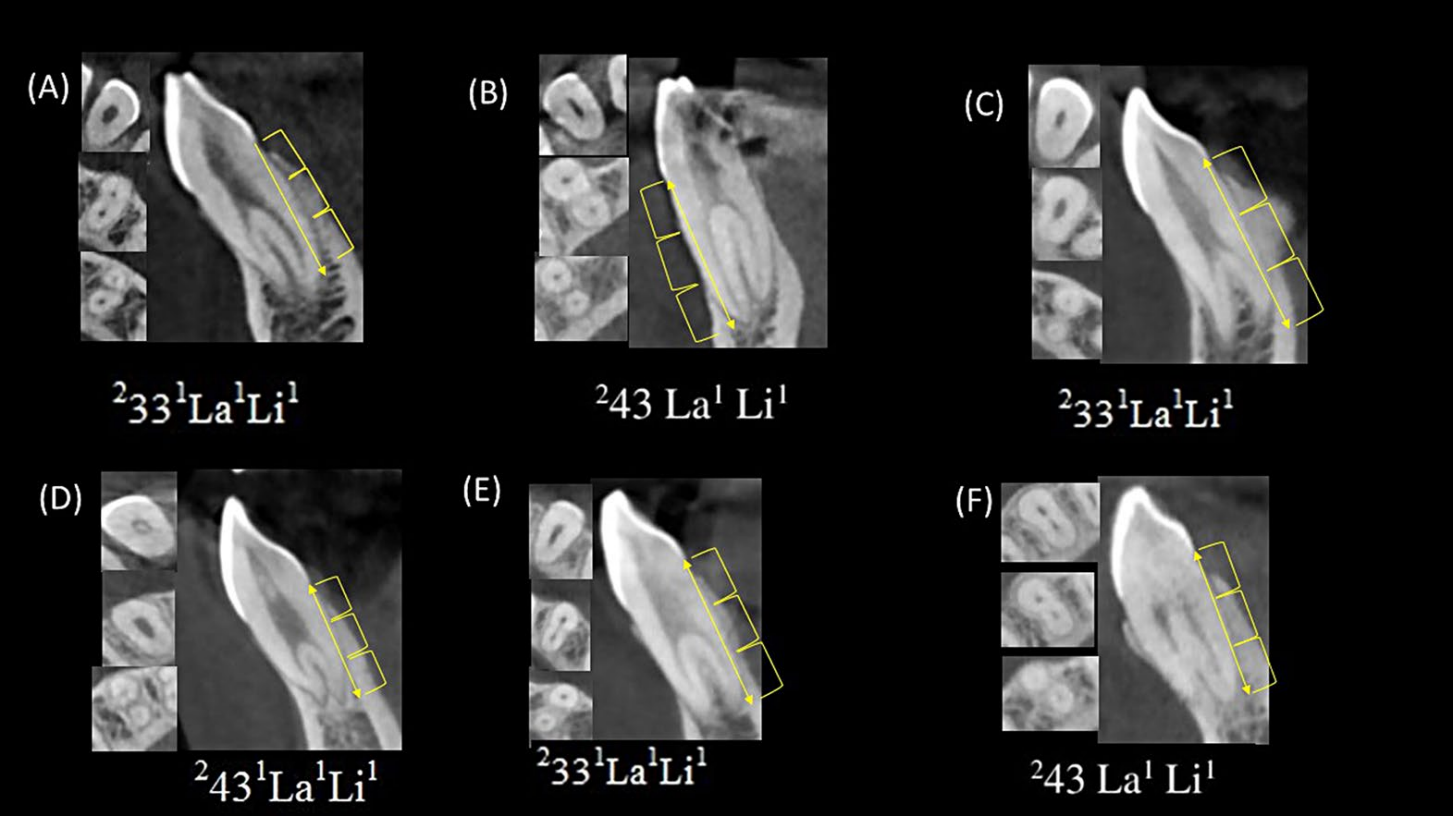
CBCT images sagittal and axial (coronal, middle and apical) views of mandibular canines showing two roots with variable levels for roots and canals splitting. A line is drawn from the CEJ to the root apex and the distance is divided into thirds. If the canal bifurcation occured in the middle or apical third then a superscript no “1” is added after the tooth number as displayed in the images (**A** to **E**)

In terms of Ahmed classification, ^1^MI^1−1^ (75.9%, *N* = 2538), ^1^MI^1–2−1^ (20.6%, *N* = 688), and ^1^MI^2−1^ (1.1%, *N* = 37) exhibited similar characteristics with Type I, III, and IV, which corresponds to Vertucci classification. Whereas, the frequencies of ^1^MI^1–2^ and ^1^MI^2−1−2−1^ were 0.3% (*N* = 10) and 0.9% (*N* = 31), respectively. The total frequencies of ^2^MI, which has no correspondence in the Vertucci classification, was 1.1% (*N* = 38). It was significantly higher in canines than in other teeth (*p* < 0.001) (Table 1; Fig. 4).

There was no significant difference between genders in the central and lateral incisors (*p* > 0.05), but significant difference was found in the canines (*p* < 0.05), Type I was found to be higher in males. The incidence of 2 roots (^2^MI by Ahmed classification) that could not be classified by Vertucci was higher in females (Table 2).

**Table 2:** The frequency of root canal configuration according to gender

	Cental Incisors		Lateral Incisors		Canines	
	M (*N* = 1554)	F (*N* = 1788)	M (*N* = 1554)	F (*N* = 1788)	M (*N* = 518)	F (*N* = 596)
Vertucci classification						
I	351 (67.8%)	436 (73.2%)	353 (68.1%)	427 (71.6%)	473 (91.3%)	499 (83.7%)
II	4 (0.8%)	4 (0.7%)	2 (0.4%)	5 (0.8%)	5 (1%)	17 (2.9%)
III	152 (29.3%)	148 (24.8%)	156 (30.1%)	154 (25.8%)	30 (5.8%)	47 (7.9%)
IV	0 (0%)	0 (0%)	1 (0.2%)	2 (0.3%)	8 (1.5%)	26 (4.4%)
V	3 (0.6%)	0 (0%)	2 (0.4%)	1 (0.2%)	2 (0.4%)	3 (0.5%)
NC	8 (1.5%)	8 (1.3%)	4 (0.8%)	7 (1.2%)	0 (0%)	4 (0.7%)
p-value	0.147^1^		0.498^1^		**0.002** ^1^	
Ahmed classification						
^1^MI^1−1^	351 (67.8%)	436 (73.2%)	353 (68.1%)	426 (71.5%)	473 (91.3%)	499 (83.7%)
^1^MI^1–2^	3 (0.6%)	0 (0%)	2 (0.4%)	1 (0.2%)	2 (0.4%)	2 (0.3%)
^1^MI^1–2−1^	152 (29.3%)	148 (24.8%)	156 (30.1%)	155 (26%)	30 (5.8%)	47 (7.9%)
^1^MI^2−1^	4 (0.8%)	4 (0.7%)	2 (0.4%)	5 (0.8%)	5 (1%)	17 (2.9%)
^1^MI^2−1−2−1^	8 (1.5%)	8 (1.3%)	4 (0.8%)	7 (1.2%)	0 (0%)	4 (0.7%)
^2^MI	0 (0%)	0 (0%)	1 (0.2%)	2 (0.3%)	8 (1.5%)	27 (4.5%)
p-value	0.147^1^		0.525^1^		**0.001** ^1^	

^1^ Chi-square test

There were significant differences between different age groups in central and lateral incisors (*p* < 0.05), but no significance was found in canines (*p* > 0.05). Type I which corresponds to 1MI^1 − 1^ of Ahmed classification was found to be higher in those older than 35 years in the central and lateral incisors (Table 3).

**Table 3:** The frequency of root canal configuration according to age

	Cental Incisors		Lateral Incisors		Canines	
	≤ 36 (*N* = 536)	> 36 (*N* = 578)	≤ 36 (*N* = 536)	> 36 (*N* = 578)	≤ 35 (*N* = 536)	> 35 (*N* = 578)
Vertucci classification						
I	347 (64.7%)	440 (76.1%)	348 (64.9%)	432 (74.7%)	471 (87.9%)	501 (86.7%)
II	3 (0.6%)	5 (0.9%)	3 (0.6%)	4 (0.7%)	14 (2.6%)	8 (1.4%)
III	169 (31.5%)	131 (22.7%)	172 (32.1%)	138 (23.9%)	33 (6.2%)	44 (7.6%)
IV	0 (0%)	0 (0%)	3 (0.6%)	0 (0%)	15 (2.8%)	19 (3.3%)
V	3 (0.6%)	0 (0%)	2 (0.4%)	1 (0.2%)	2 (0.4%)	3 (0.5%)
NC	14 (2.6%)	2 (0.3%)	8 (1.5%)	3 (0.5%)	1 (0.2%)	3 (0.5%)
p-value	**< 0.001** ^1^		**0.005** ^1^		0.517^1^	
Ahmed classification						
^1^MI^1−1^	347 (64.7%)	440 (76.1%)	347 (64.7%)	432 (74.7%)	471 (87.9%)	501 (86.7%)
^1^MI^1–2^	3 (0.6%)	0 (0%)	2 (0.4%)	1 (0.2%)	1 (0.2%)	3 (0.5%)
^1^MI^1–2−1^	169 (31.5%)	131 (22.7%)	173 (32.3%)	138 (23.9%)	33 (6.2%)	44 (7.6%)
^1^MI^2−1^	3 (0.6%)	5 (0.9%)	3 (0.6%)	4 (0.7%)	14 (2.6%)	8 (1.4%)
^1^MI^2−1−2−1^	14 (2.6%)	2 (0.3%)	8 (1.5%)	3 (0.5%)	1 (0.2%)	3 (0.5%)
^2^MI	0 (0%)	0 (0%)	3 (0.6%)	0 (0%)	16 (3%)	19 (3.3%)
p-value	**< 0.001** ^1^		**0.004** ^1^		0.439^1^	

^1^ Chi-square test

A moderate correlation was found between the left and right sides in all tooth types and in both classifications. The correlation, according to Cramer’s V analysis, was highest in the central incisor (V = 0.46) with a similarity of 75%, and it was the lowest in the lateral incisor (V = 0.38) with a similarity of 67% (Table 4).

**Table 4:** Cramer’s V values that indicate bilateral relationship between left and right teeth

Tooth type	Vertucci classification		Ahmed classification	
	Similarity	Cramer’s V	Similarity	Cramer’s V
Cental Incisors	2518 (75.45%)	0.46 **	2517 (75.42%)	0.46 **
Lateral Incisors	2254 (67.54%)	0.38 **	2252 (67.48%)	0.38 **
Canines	2166 (64.90%)	0.45 **	2164 (64.84%)	0.45 **

* >0.10 weak; ** >0.30 moderate; *** >0.50 strong correlation

There was a significant difference among tooth types regarding the bifurcation levels (*p* < 0.001). In all tooth types, the bifurcation level was mostly in the middle area followed by coronal then apical (Table 5). 75.9% (*N* = 2538) of the teeth and particularly the canines did not exhibit any divergence/merging in total, the middle/apical divergence/merging was the most common level in centrals and laterals compared to canines *p* < 0.001) (Table 6).

**Table 5:** The frequency of bifurcation level according to tooth type

Bifurcation level	Cental Incisors	Lateral Incisors	Canines
None	972 (70.64%)	779 (69.92%)	787 (87.25%)
Coronal	29 (2.78%)	27 (2.42%)	31 (2.6%)
Middle	112 (26.03%)	304 (27.28%)	290 (10.05%)
Apical	1 (0.53%)	4 (0.35%)	6 (0.08%)
Total	1114 (100%)	1114 (100%)	1114 (100%)
p-value	< 0.001^1^		

^1^ Chi-square test

**Table 6:** The frequency of divergence/merging levels according to the tooth type

Divergence/merging level	Central (*N* = 1114)	Lateral (*N* = 1114)	Canine (*N* = 1114)	Total (*N* = 3342)
Coronal/apical	24 (2.2%)	16 (1.4%)	9 (0.8%)	49 (1.5%)
Middle/apical	250 (22.4%)	250 (22.4%)	36 (3.2%)	536 (16%)
Middle/middle	40 (3.6%)	54 (4.8%)	76 (6.8%)	170 (5.1%)
Apical/apical	6 (0.5%)	4 (0.4%)	1 (0.1%)	11 (0.3%)
Coronal/middle	4 (0.4%)	8 (0.7%)	2 (0.2%)	14 (0.4%)
Coronal/coronal	3 (0.3%)	3 (0.3%)	18 (1.6%)	24 (0.7%)
None	787 (70.6%)	779 (69.9%)	972 (87.3%)	2538 (75.9%)
p-value	< 0.001^1^			

^1^ Chi-square test

## Discussion

Knowledge of the anatomical configurations of the roots and canals of each tooth is crucial in order to predict possible complications during treatment. Although the MDA has a single root and canal in most cases [19–22], clinicians should consider possible variations to remove the pulp tissue and necrotic debris effectively without negatively affecting the structural integrity of the tooth and root.

Consistent with previous studies [19, 23], Type I Vertucci (Equivalent to ^1^MI^1−1^ in Ahmed classification) was the most common type of canal morphology in MDI. The second most common type of root canal configuration for MDI was Type III Vertucci (Equivalent to to ^1^MI^1–2−1^), consistent with several studies [16, 21, 22]. On the other hand, in the Malaysian population Type III Vertucci (Equivalent to ^1^MI^1–2−1^) was the most common root canal configuration for MDI followed by Type I Vertucci (Equivalent to ^1^MI^1−1^) [6].

Root canal configurations in 16 (1.4%) mandibular centrals and 11 (1%) mandibular laterals teeth could not be classified with Vertucci’s classification. The correspondence of all these in Ahmed classification was ^1^MI^2−1−2−1^. This was also the third most common type of root canal configuration for MDI according to Ahmed classification.

In this study, it was found that approximately 30% of MDA has two canals. It has been reported that the second canal (lingual) is mostly missed by clinicians [24]. This is probably because the dentist cannot recognize the presence of the lingual canal, clinically it is recommended to extend the accessl cavity to the incisal edge in the form of an oval preparation with a mesiodistal width of less than 2 mm to locate the lingual canal [24, 25]. Considering that the lingual canal merges into the labial canal before the apex and ends in a single canal, this may not compromise the outcome of endodontic treatment and the complete filling of the labial canal in the apical third [16].

Although several studies reported that MDA are single-rooted [20, 21, 26], in this study there were two-rooted laterals (0.3%) and canines (3.1%) similar to the study by Zhengyan, et al. [27]. The frequency of two-rooted mandibular canines was 0.3–1.9% in previous studies [6, 28, 29], compared to 3.1% in this study.

In a study of anterior teeth in a Jordanian subpopulation using canal staining and root-clearing technique, the frequency of Type 1 Vertucci (73.8%) was close to that of the present study (76%) [19]. However, different frequencies have been found for other types, possibly due to differences in the methods used. Studies have demonstrated the high reliability of CBCT in detecting root canal morphology compared to visual inspection [30, 31]. The European Society of Endodontology position statement on CBCT imaging recommends that CBCT should be used to assess complex root canal morphology [32].

According to the present study, Type I (^1^ MI ^1−1^) and Type III (^1^ MI ^1–2−1^) were found to be the most common in MDC, accounting for 90.7% and 8.2%, respectively. Several studies carried out in Iran (97.6%) [28], Israel (89.7%) [29], Portugal (90.2%) [23], Brazil (90.5%) [4], and Malaysia (87%) [6] have reported a similar frequency of Type I Vertucci. In the study by Karobari, et al. on Malaysian population [6], they came across two MDC morphologies (^1^MD ^2−1−2−1−2−1^ and ^1^MD ^2−1−2−1^) with a 0.001% rate which could not be categorized based on the Vertucci system. In this study, only one type of MDC morphology (^1^MI^2−1−2−1^) was found that could not be classified by Vertucci with a 0.4% occurrence rate.

This study, in line with the research of Geduk, et al. [33], found no significant gender-related differences in the root canal morphology of MDI. The only noticeable distinction between genders was found in MDC, the variation in root canal morphology of MDC was more diverse in females than in males. Similar to Karobari, et al. [6], females had an extra ^1^MI^2−1−2−1^ classification. Although some studies suggest that males have greater variation in canals, others found no significant difference [3, 33]. The difference in the results of studies could be attributed to factors such as sample size, methodology, or genetic traits of the individuals involved in the study.

Consistent with previous studies it seems that age plays a significant role in determining root canal morphology in MDI. There was a greater range of root canal variations among younger patients [6, 27]. As we age, teeth naturally undergo tertiary dentinogenesis. This involves the deposition of dentin in response to injury, either by odontoblasts or odontoblast like cells from the pulp, depending on the extent of the injury. As a result pulp volume decreases [34] and the root canal may transform from a complex to a simpler configuration with age [27].

Although Vertucci classification [7] is widely used to categorize root canal morphology, it fails to consider the number of roots present in anterior and premolar teeth. Bi-rooted teeth in the anterior and premolars are classified as type IV or type V, which may mislead the clinician during root canal treatment procedures [18]. The classification by Ahmed [8] now classifies bi-rooted anterior and premolars using a single code that takes into account the number of roots and canal morphology. In complex canal variations, there is no need to memorize Roman numbers of classifications like Vertucci system. According to a survey of senior dental students in Malaysia, more than 90% of the students found the new system to be more precise and convenient compared to Vertucci classification [35].

A recent systematic review included 15 studies that compared the Ahmed et al. system with the Vertucci classification. The results revealed that both system were able to classify simple canal configurations in single-rooted anterior teeth; however Ahmed et al. system provided more accurate and comprehensive categorization of single-rooted teeth with complex anatomy [36]. For CBCT studies on the antomy of mandibular anterior teeth, up to 2.2% of the included sample were categorized as non-classifiable using the Vertucci System [6], and this percentage was more evident up to 6.6% in a micro CT evaluation study [16].

In the analysis using Cramer’s V, there was a moderate correlation observed between the canal morphologies on the left and right sides. According to Lin, et al. [37], there is a 92.7% and 89.2% similarity in morphology between the left-right sides for MDC and MDL, respectively. In this study, even though lower values were obtained (MDS:75.42%, MDL: 67.48%, MDC: 64.84%), this correlation was noticable, thus when dealing with mandibular anterior teeth, it is important for the clinicians to take into account their morphological similarity.

The bifurcation of a root canal refers to the point where a single canal splits into two smaller ones. Previously, dentists relied on periapical radiographs to detect the presence of bifurcations through the “fast break” guideline, where the root canal suddenly narrows or even disappears [1, 38]. CBCT images are now capable of visualizing the root canal bifurcation and supplying data for quantitative evaluation as well [30, 38]. Martins, et al. reported that the main root canal can merge and split at any level in the root [17]. While in this study the middle third of the MDA had the highest number of bifurcations, which is similar to the findings by a previous study on MDI [37]. Clinicians should consider the possibility of second canal at the mid root level, inspection under magnification and the use of CBCT whenever needed is recomended to avoid potential complications.

The reliability of CBCT imaging depends greatly on the voxel size, with smaller size leading to better results. In this study, the CBCT voxel size used was 150 μm, which is higher than that of micro-CT systems but still reliable in identifying the number of root canals and specific root anatomy. While micro-CT can achieve voxel sizes as small as 5 μm, CBCT devices with smaller fields of view may reach up to 76 μm. Hatipoğlu, et al. reported that the voxel size (above or below 150 μm) did not affect the detection of the midle mesial and distolingual canals in mandibular first molars [39, 40].

## Conclusions

1. While single canal was the most common configuration in MDA of a Jordanian population (76%), variation in the morphology was still present in 24% of the cases particualrly in males, lateral incisors and canines. This requires particular attention and the use of magnification during endodontic treatment, considering the level of divergence of canals was mostly in the middle third of the root.
2. Ahmed classification was a simple classification system that provides information on tooth number, number of roots and details on canal configuration in a single code.

## Data Availability

The data that support the findings of this study are available from the corresponding author upon reasonable request.
